# *Pseudogymnoascus destructans* transcriptome changes during white-nose syndrome infections

**DOI:** 10.1080/21505594.2017.1342910

**Published:** 2017-07-13

**Authors:** Sophia M. Reeder, Jonathan M. Palmer, Jenni M. Prokkola, Thomas M. Lilley, DeeAnn M. Reeder, Kenneth A. Field

**Affiliations:** a Department of Biology, Bucknell University, Lewisburg, PA, USA; b Center for Forest Mycology Research, Northern Research Station, US Forest Service, Madison, WI, USA

**Keywords:** dual RNA-Seq, fungal virulence, host-pathogen interactions, transcriptomics

## Abstract

White nose syndrome (WNS) is caused by the psychrophilic fungus *Pseudogymnoascus destructans* that can grow in the environment saprotrophically or parasitically by infecting hibernating bats. Infections are pathological in many species of North American bats, disrupting hibernation and causing mortality. To determine what fungal pathways are involved in infection of living tissue, we examined fungal gene expression using RNA-Seq. We compared *P. destructans* gene expression when grown in culture to that during infection of a North American bat species, *Myotis lucifugus*, that shows high WNS mortality. Cultured *P. destructans* was grown at 10 to 14 C and *P. destructans* growing *in vivo* was presumably exposed to temperatures ranging from 4 to 8 C during torpor and up to 37 C during periodic arousals. We found that when *P. destructans* is causing WNS, the most significant differentially expressed genes were involved in heat shock responses, cell wall remodeling, and micronutrient acquisition. These results indicate that this fungal pathogen responds to host-pathogen interactions by regulating gene expression in ways that may contribute to evasion of host responses. Alterations in fungal cell wall structures could allow *P. destructans* to avoid detection by host pattern recognition receptors and antibody responses. This study has also identified several fungal pathways upregulated during WNS infection that may be candidates for mitigating infection pathology. By identifying host-specific pathogen responses, these observations have important implications for host-pathogen evolutionary relationships in WNS and other fungal diseases.

## Introduction

Fungal pathogens have emerged as major threats to biodiversity^1^ and human health.^2^ The diversity of these infectious eukaryotes and their hosts present new challenges in characterizing the interactions between host, pathogen, and the environment that lead to pathogenesis. One successful approach is to use systems biology to compare whole-transcriptome changes in gene expression between the pathogen infecting the host, the host without the pathogen, and the pathogen without the host.^3-5^ This dual RNA-Seq approach can be used to identify genetic factors from the pathogen that contribute to host colonization and manipulation of host-pathogen interactions.

Among fungal emerging infectious diseases, white-nose syndrome (WNS) in bats has spread from Eurasia, where it is endemic, to North America,^6-8^ where it is decimating several species of hibernating bats. Susceptible species, such as the little brown myotis (*Myotis lucifugus*) have shown population declines up to 90% in affected hibernacula.^9-11^ WNS is caused by *Pseudogymnoascus destructans*, a psychrophilic fungus that grows in cold hibernacula and causes cutaneous infections in bats while they hibernate. During WNS, *P. destructans* invades the skin tissue, forming subcutaneous lesions identified as cupping erosions by histopathology.^12^ The infection disrupts the hibernation behavior of susceptible bats and leads to more frequent arousals from torpor, premature energy depletion, electrolyte imbalance, and death.^13-16^

WNS does not affect all species of bats equally. Many, but not all, North American species are being severely affected,^17,18^ while most European bats can host *P. destructans* infections, but have low mortality from WNS.^19-22^ Coevolution of *P. destructans* and Eurasian bats, such as Daubenton's myotis (*M. daubentonii*), appears to have adapted these populations to a commensal or parasitic relationship with lower pathology.^8^ North American bats, on the other hand, have yet to benefit from such selection against extirpation of the host species^23^ and some species face the possibility of regional extinctions.^10,18,24^ The virulence of the *P. destructans* infection is controlled by a combination of the environment (i.e., temperature and humidity of the hibernaculum), the host (and the host's response to infection), and the pathogen (and the pathogen's response to the host).^25^ In this study, we focus on the third component of this epidemiological triangle by dissecting the genetic components that allow *P. destructans* to infect hosts and become a virulent pathogen.

Whether *P. destructans* remains a commensal parasite or becomes pathogenic is determined by host-pathogen interactions.^8,26^ We have previously examined the host response of the WNS-susceptible *M. lucifugus* to *P. destructans* infection in the wing membrane and found robust gene expression changes in the host during hibernation.^27^ We now shift our focus to characterize previously hypothesized virulence attributes of the fungus that include immune evasion, nutrient acquisition, stress responses, and tissue invasion.^28,29^ We measured *P. destructans* gene expression at the whole-transcriptome level, comparing expression patterns between the fungus when growing in culture and when infecting a North American bat species.

## Results

Two different groups of samples were used to measure gene expression in *P. destructans* for this study (Table 1). Gene expression during infection of *M. lucifugus* was measured in the MyLu samples of wing tissue from 6 individual *P. destructans-* infected *M. lucifugus* collected 60–120 minutes after arousal from hibernation in caves in Kentucky, USA. Gene expression in *P. destructans* during infection was compared with 4 samples from the 20631–21 strain of *P. destructans* growing in culture at 10–14°C for 23 d on Sabouraud dextrose agar plates (Table 1).

**Table 1.** RNA-Seq data sets used for analysis and RSEM expected counts.

Group	Sample	SRA Accession	Sequencing	Reads post-trim	*P. destructans* counts	*M. lucifugus* counts	Percent *Pd*
MyLu	KYMYLU06W	SRR1916825	PE 101 bp	19 289 825	99 055	5 190 125	1.9%
	KYMYLU07W	SRR1916826	PE 101 bp	18 862 520	121 838	5 379 370	2.2%
	KYMYLU11W	SRR1916827	PE 101 bp	19 302 516	98 878	5 034 500	1.9%
	KYMYLU19W	SRR1916842	PE 101 bp	17 642 460	123 139	4 535 156	2.6%
	KYMYLU23W	SRR1916830	PE 101 bp	14 997 956	85 249	3 599 787	2.3%
	KYMYLU39W	SRR1916832	PE 101 bp	17 609 994	59 888	4 252 402	1.4%
Culture	SRR1270148		PE 50 bp	22 792 423	13 820 072		
	SRR1270408		PE 50 bp	24 400 308	15 080 834		
	SRR1270412		PE 101 bp	107 250 955	63 836 991		
	SRR1270417		SE 51 bp	27 402 575	18 301 930		

### Comparison of infected and uninfected bats

Prior to comparing the expression of *P. destructans* genes during host infection to those in culture, we confirmed that infection levels in host tissues were sufficient to measure pathogen gene expression by quantifying the number of RNA-Seq reads that mapped to the *P. destructans* transcriptome (Table S1). Compared to a group of samples from *M. lucifugus* not infected with *P. destructans* (Figure S1), the samples from the infected bats from Kentucky showed significantly higher levels of *P. destructans* transcripts (t = 8.84, p < 0.00001). In the wing samples from infected bats, we found that 5990 ± 324 *P. destructans* genes were expressed at a minimum count of 1, representing 63% of all *P. destructans* genes (Table S1). These samples expressed 13 512 ± 357 *M. lucifugus* genes, representing 52% of all bat genes. Using a minimum of 1 count in any sample, the cultured samples expressed 8825 genes and the wing samples expressed 7264 genes of 9575 known *P. destructans* genes (Table S1). These results indicate that sufficient read depth was obtained in this data set to measure *P. destructans* gene expression, at least for the majority of genes.

### Comparison of *P. destructans* gene expression during WNS and culture

**Figure 1.**
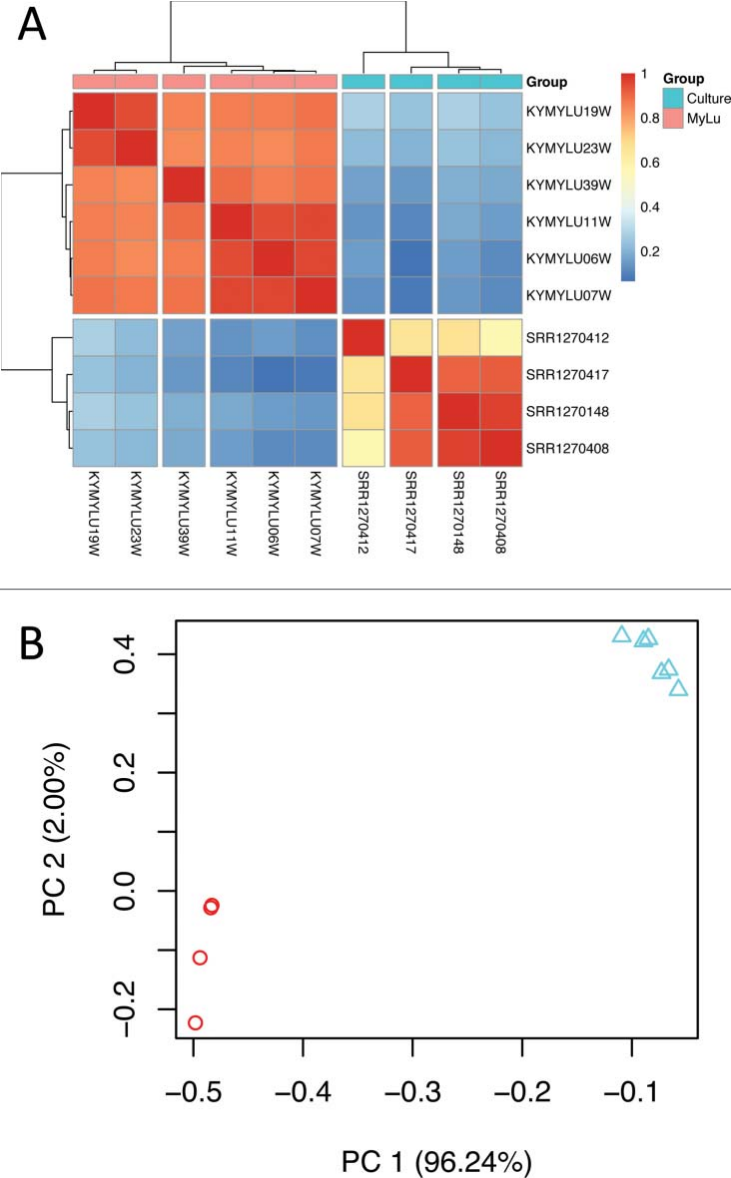
Gene expression of *P. destructans* in culture and when infecting *M. lucifugus*. (a) Hierarchical clustering of differentially expressed *P. destructans* trimmed mean of M-values (TMM)-normalized gene expression levels using Pearson correlation complete-linkage clustering with Euclidean distances. Scale shows Pearson correlation coefficient. Vertical breaks in the heatmap indicate clustering supported by bootstrap analysis at a confidence of 99% and the horizontal break indicates separate clustering of the different groups of samples. (b) Principal component analysis of global *P. destructans* gene expression using log2-transformed TMM-normalized expression levels. The principal components PC1 and PC2 represent 96% and 2% of the variance in the data, respectively. Triangles represent the MyLu samples and circles represent the culture samples.

Using both hierarchical clustering (Fig. 1A) and principal component analysis (Fig. 1B), we found that the patterns of *P. destructans* gene expression were similar in each group of samples (cultured or WNS). We observed a small batch effect between the cultured samples that were grown at different times and sequenced differently (Table 1). We also found that samples from bats KY19 and KY23, which came from a different cave in the same county as bats KY06, KY07, and KY11,^27^ clustered separately from these samples and from sample KY39, which came from a different county. These results suggest that some of the differences in gene expression that we observe within the 2 groups could be due to variations in the environmental conditions or genetic differences between the *P. destructans* isolated growing in different hibernacula. However, the largest differences appear to be due to the different growth conditions between culture and growth on bats.

**Figure 2.**
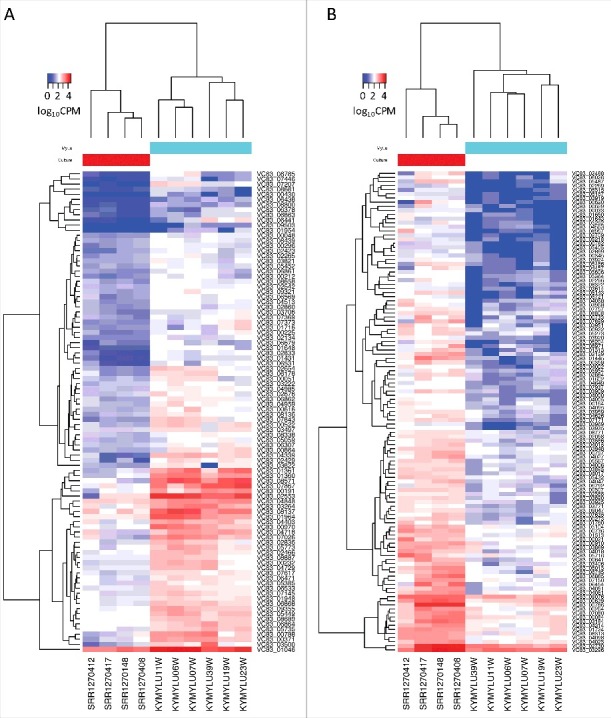
Expression levels of differentially expressed *P. destructans* genes. Heatmaps show the expression level in counts per million (CPM) of (a) the 94 *P. destructans* genes upregulated in the MyLu samples compared with the Culture samples or (b) the 117 genes upregulated in the Culture samples compared with the MyLu samples. Genes were identified as differentially expressed (FDR < 0.001) by both edgeR and DESeq2 and expressed (CPM > 0) in at least 2 of the MyLu samples. The scale is log_10_ CPM with a maximum of 4.5 (a) or 4.1 (b).

**Figure 3.**
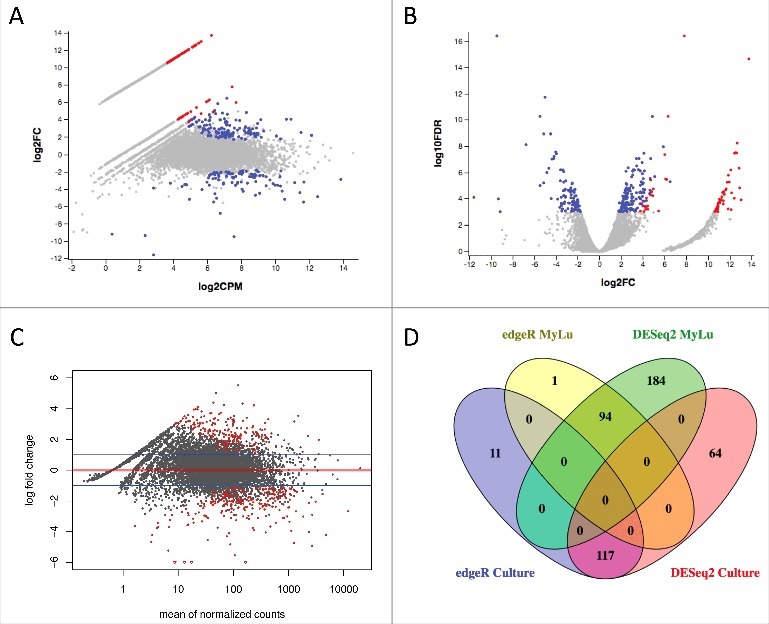
Differential *P. destructans* gene expression in culture and when infecting *M. lucifugus*. **(a)** Expression of *P. destructans* genes is compared by edgeR between culture and *M. lucifugus* infection with an MA plot. The mean expression level (log_2_ counts per million (CPM)) and the fold change (log_2_ FC) are shown for each gene. Genes more highly expressed in culture are on the upper half of the graph and those more highly expressed in *M. lucifugus* tissue in the lower half. Blue points indicate differential expression (FDR ≤ 0.001 determined by edgeR) that are expressed in at least 2 MyLu samples. Red points indicate significant differential expression for genes that were not expressed in at least 2 MyLu samples. An interactive version of this graph is available as Data Set S2. After unzipping File S2 and opening the html file in a web browser, hover over each point to view the annotation metadata for that gene and the expression level (in log_2_CPM) for each sample. Individual genes can be found by searching, for example by entering VC83_01361 in the search box. **(b)** Expression of *P. destructans* genes is compared by edgeR between culture and *M. lucifugus* infection with a volcano plot. The fold change (log_2_) and the FDR (log_10_) are shown for each gene. Genes more highly expressed in culture are on the right half of the graph and those more highly expressed in *M. lucifugus* tissue in the left half. Blue points indicate differential expression (FDR ≤ 0.001 determined by edgeR), with colors as for (a). An interactive version of this graph is available as Data Set S3 and can be manipulated as described above. **(c)** Expression of *P. destructans* genes is compared by DESeq2 between culture and *M. lucifugus* infection with an MA plot. The mean expression level and the fold change (log_2_) are shown for each gene. The red line indicates equal expression and the blue line indicate a 2-fold change. Genes more highly expressed in culture are on the upper half of the graph and those more highly expressed in *M. lucifugus* tissue in the lower half. Red points indicate differential expression (FDR ≤ 0.001 determined by DESeq2). **(d)** A Venn diagram compares the number of *P. destructans* genes identified as differentially expressed by edgeR and DESeq2. The number of genes shared by edgeR and DESeq2, or unique to each method, are shown using a maximum FDR of 0.001 and minimum fold change of 2 for genes upregulated in *M. lucifugus* infections or upregulated in culture after removing genes not expressed in at least 2 of the MyLu samples. Table S2 lists results for all *P. destructans* genes.

We then compared *P. destructans* gene expression during WNS infection of *M. lucifugus* to the 20631–21 strain of *P. destructans* grown in culture using both edgeR (Fig. 2, 3A, 3B) and DESeq2 (Fig. 3C). Because of the lower depth of sequencing for the WNS samples, we then filtered the results to exclude any *P. destructans* genes that were not expressed in at least 2 of the 6 MyLu samples. With a cutoff of 0.001 for FDR and a 2-fold minimum change, similar results were obtained using these 2 different analysis methods (Fig. 3D), with the majority of the genes identified as differentially expressed by edgeR also being identified by DESeq2.

Using the subset of genes identified by both edgeR and DESeq2, 94 *P. destructans* genes were identified as more highly expressed during WNS infection of *M. lucifugus*, and 117 genes were more highly expressed in *P. destructans* growing in culture (Fig. 3D, Table S2). Using our Trinotate annotation, we identified 39 genes that showed significant changes in expression during WNS whose putative functions could contribute to virulence by affecting tissue invasion, the heat shock response, nutrient acquisition, immune evasion, and other pathways (Table 2).

**Table 2.** Selected *P. destructans* genes differentially expressed between culture and WNS-affected *M. lucifugus* that have putative functions implicated in fungal virulence.

			edgeR		DESeq2		
Gene^a^	Full Name	BLASTX^b^	FC^c^	FDR^d^	Cult^e^	WNS^e^	FDR^f^
Secreted Enzymes							
VC83_01361	Major allergen Asp f 2	ALL2_ASPFU	36.0	1.11E-09	53.2	2044.8	7.80E-16
VC83_00616	Lipase 1	LIP1_GEOCN	9.1	1.84E-05	31.3	290.5	1.27E-07
Heat Shock Response							
VC83_02553	30 kDa heat shock protein	HSP30_NEUCR	29.4	9.69E-07	249.4	7737.6	3.63E-09
VC83_07843	Hsp70 nucleotide exchange factor FES1	FES1_NEUCR	19.0	8.14E-08	19.1	378.4	9.52E-12
VC83_00970	Heat shock protein 78, mitochondrial	HSP78_SCHPO	12.4	4.17E-06	126.8	1643.3	1.15E-08
VC83_00522	Protein psi1	PSI1_SCHPO	9.9	5.21E-06	42.2	431.6	1.90E-08
VC83_01964	Heat shock protein hsp88	HSP88_NEUCR	9.5	2.15E-05	291.4	2874.6	2.03E-07
VC83_08137	Heat shock protein hsp98	HSP98_NEUCR	9.0	7.52E-05	449.0	4178.2	3.60E-06
VC83_01046	Heat shock 70 kDa protein 2	HSP72_PARBA	7.3	2.04E-04	2430.7	18 564	8.05E-06
VC83_02466	Uncharacterized protein C1711.08	YNY8_SCHPO	5.3	1.41E-04	104.3	571.7	2.61E-07
VC83_08187	Heat shock protein 82	HSP82_AJECA	4.3	1.91E-03	989.3	4490.9	7.48E-05
VC83_09034	Unchar. J domain-containing protein C63.13	YCJD_SCHPO	4.3	3.20E-03	61.6	272.2	6.18E-04
VC83_06435	Heat shock protein sti1 homolog	STI1_SCHPO	4.1	2.03E-03	192.3	830.4	6.31E-05
Ion Homeostasis							
VC83_01360	Zinc-regulated transporter 1	ZRT1_YEAST	18.6	5.62E-08	91.1	1801.3	1.61E-12
VC83_07026	Calcium-transporting ATPase 3	ATC3_SCHPO	11.7	2.53E-04	125.6	1481.6	7.19E-05
VC83_00191	Putative Copper transporter protein (PFAM)		10.3	2.37E-05	112.5	1225.7	6.78E-07
VC83_06862	Calcium-transporting ATPase 3	ATC3_SCHPO	6.2	4.74E-05	36.3	231.6	9.40E-07
VC83_01014	Calcium-transporting ATPase 2	ATC2_SCHPO	3.4	2.64E-03	193.9	698.9	5.31E-06
VC83_04094	Metal homeostasis factor ATX1	ATX1_YEAST	−3.6	4.37E-03	87.6	25.1	1.06E-03
VC83_00736	Na(+)/H(+) antiporter 1	NAH1_ZYGRO	−6.2	4.25E-07	522.8	88.4	1.23E-09
Cell Wall Remodeling							
VC83_03500	Spherulin-1A	SR1A_PHYPO	22.2	9.41E-05	31.4	758.3	6.99E-06
VC83_07867	Uncharacterized protein AFUA_6G02800	YA280_ASPFU	21.2	8.97E-08	169.0	3783.0	5.93E-11
VC83_00788	Endochitinase 1	CHI1_APHAL	11.6	2.07E-04	106.9	1327.3	1.93E-05
VC83_07327	Probable glucan endo-1,3-β-glucosidase eglC	EGLC_NEOFI	6.5	1.93E-03	221.5	1489.4	4.95E-04
VC83_04729	Endochitinase 1	CHI1_COCIM	6.3	8.24E-05	83.2	553.7	5.69E-07
VC83_07145	Mannan endo-1,6-α-mannosidase DCW1	DCW1_YEAST	5.9	2.51E-05	74.0	450.7	7.40E-09
VC83_05104	Chitin synthase 4	CHS4_NEUCR	−3.4	8.74E-04	397.1	121.7	1.56E-05
VC83_09076	Glucan 1,3-β-glucosidase	EXG1_COCCA	−3.5	1.99E-04	1722.2	512.6	6.20E-10
VC83_00261	Mannan endo-1,6-α-mannosidase DFG5	DFG5_CANAL	−3.8	3.97E-03	145.4	39.1	3.12E-03
VC83_08448	Protein SUR7	SUR7_CANAL	−10.1	1.43E-03	726.2	71.1	5.55E-03
VC83_05292	Cell wall mannoprotein CIS3	CIS3_YEAS7	−14.8	3.30E-05	97.9	6.8	1.76E-04
VC83_01650	Mannan endo-1,6-α-mannosidase DCW1	DCW1_YEAST	−15.4	1.18E-06	106.0	7.0	8.53E-07
Other							
VC83_06039	Putative heme-binding peroxidase	CCPR2_ASPFU	7.5	4.90E-03	42.7	340.3	2.49E-03
VC83_00225	Putative cryptochrome DASH, mitochondrial	CRYD_NEUCR	7.5	2.15E-05	18.2	140.4	5.05E-07
VC83_06307	Squalene monooxygenase	ERG1_CANAL	4.5	2.51E-04	40.5	188.4	1.24E-07
VC83_03222	Probable GTP cyclohydrolase-2	RIB1_SCHPO	4.1	7.67E-04	44.1	189.4	2.58E-06
VC83_01624	Leptomycin B resistance protein pmd1	PMD1_SCHPO	3.8	3.55E-03	164.9	662.7	4.06E-04
VC83_06509	Thioredoxin reductase	TRXB_NEUCR	3.1	2.03E-03	49.8	163.1	8.71E-07
VC83_08771	Probable transporter MCH5	MCH5_YEAST	−4.2	2.18E-04	177.5	45.3	8.53E-07

Notes.

a *P. destructans* gene (Drees et al. 2016).

b BLAST hit with the lowest E-value in the Swissprot database. Only homologs with E < 1E-04 were considered.

c Fold change in gene expression of the WNS samples compared with the culture samples determined by EdgeR. Negative values indicate higher expression in the culture samples. Dashed lines separate genes with higher expression in WNS from genes with higher expression in culture.

d Adjusted p-value of differential expression determined by edgeR after Benjamini-Hochberg FDR correction.

e Mean normalized expression level (TPM) in culture or WNS samples determined by DESeq2.

f Adjusted p-value of differential expression determined by DESeq2 after Benjamini-Hochberg FDR correction.

We specifically examined the expression levels of secreted proteases, because they have been implicated in the pathogenesis of WNS.^30,31^ Protease genes were identified by homology and by PFAM analysis^32^ and the expression of these genes was compared in the 5 culture samples and 6 *M. lucifugus* WNS samples (Table S2). Table 3 lists selected protease genes and demonstrates that the genes for subtilase-family proteases are more highly expressed during culture than during tissue invasion. Other proteases are highly expressed during host infection, such as *VC83_01361*, the *P. destructans* homolog of the *Aspergillus fumigatus* major allergen Aspf2, show lower gene expression when *P. destructans* is growing in culture.

**Table 3.** Expression of selected *P. destructans* protease genes.

			edgeR		DESeq2		
Gene^a^	Full Name	BLASTX^b^	FC^c^	FDR^d^	Cult^e^	WNS^e^	FDR^f^
Subtilase-family Proteases							
VC83_09074	Subtilisin-like protease 3 (Destructin-3)	SUB3_PSED2	1.8	0.66	29.3	51.6	0.67
VC83_06062	Subtilisin-like protease 2 (Destructin-1)	SUB2_PSED2	−1.3	0.78	502.4	375.4	0.80
VC83_07090	Subtilisin-like protease Spm1	SPM1_MAGO7	−1.5	0.30	2056.9	1421.4	0.12
VC83_06607	Protease Kexin 2	KEX2_CANAW	−2.3	0.05	147.8	66.9	0.04
VC83_04892	Subtilisin-like protease 1 (Destructin-2)	SUB1_PSED2	−3.0	0.33	5800.6	1962.1	1.00
VC83_02181	Tripeptidyl-peptidase sed2	SED2_ASPFU	−5.5	0.0011	791.6	152.6	0.0045
Other Putative Secreted Proteases							
VC83_01361	Major allergen Aspf2	ALL2_ASPFU	36.0	1.11E-09	53.2	2044.8	7.80E-16
VC83_03800	Disintegrin and metalloprotease domain-containing protein B	ADMB_ASPFU	2.8	0.0044	94.6	277.4	1.50E-10
VC83_02385	Zinc metalloprotease ZmpB	ZMPB_STRPN	2.5	0.15	23.6	58.7	0.01
VC83_08633	Threonine aspartase 1	TASP1_HUMAN	2.2	0.27	10.4	24.2	0.14
VC83_05359	Calpain-like protease palB	PALB_EMENI	1.8	0.24	41.7	74.8	0.05
VC83_03810	Carboxypeptidase Y homolog ARB_06361	SCPE_ARTBC	1.9	0.53	11.0	20.5	0.37

a *P. destructans* gene (Drees et al. 2016).

b BLAST hit with the lowest E-value in the Swissprot database. Only proteins with E < 1E-04 were considered.

c Fold change in gene expression of the WNS samples compared with the culture samples determined by EdgeR. Negative values indicate higher expression in the culture samples. A dashed line separate genes with higher expression in WNS from genes with higher expression in culture.

d Adjusted p-value of differential expression determined by edgeR after Benjamini-Hochberg FDR correction.

e Mean normalized expression level (TPM) in culture or WNS samples determined by DESeq2.

f Adjusted p-value of differential expression determined by DESeq2 after Benjamini-Hochberg FDR correction.

To further explore the functional pathways that regulate infection, gene ontology enrichment analysis was performed using the genes identified by edgeR at a maximum FDR of 0.05 and minimum fold-change of 2. We examined the annotated functions of *P. destructans* genes upregulated in either *M. lucifugus* infections or in culture (Table 4). This analysis determined that several pathways involved in peptide and nitrogen metabolism are significantly enriched in *P. destructans* during infection (FDR < 0.05). While growing in culture, *P. destructans* showed enrichment of oxidation-reduction and transport pathways (FDR < 0.001) and depletion of other metabolic pathways (FDR < 0.05).

**Table 4.** Gene ontology analysis of *P. destructans* pathways altered during WNS.

GO Category	Biological Process	E/P^1^	Ratio in study^2^	p	FDR^3^
Upregulated during WNS infection					
GO:0006518	peptide metabolic process	e	23/410	2.49E-08	<0.001
GO:0006412	translation	e	21/410	9.07E-08	<0.001
GO:0043043	peptide biosynthetic process	e	21/410	1.90E-07	<0.001
GO:0043603	cellular amide metabolic process	e	23/410	5.57E-07	0.002
GO:0042254	ribosome biogenesis	e	8/410	1.04E-06	0.002
GO:0043604	amide biosynthetic process	e	21/410	1.58E-06	0.002
GO:0022613	ribonucleoprotein complex biogenesis	e	8/410	2.59E-06	0.002
GO:0044085	cellular component biogenesis	e	8/410	1.16E-05	0.008
GO:0034645	cellular macromolecule biosynthetic process	e	28/410	2.62E-05	0.012
GO:1901566	organonitrogen compound biosynthetic process	e	31/410	2.82E-05	0.012
GO:0044271	cellular nitrogen compound biosynthetic process	e	34/410	9.20E-05	0.028
Upregulated in culture					
GO:0055114	oxidation-reduction process	e	75/846	2.42E-07	<0.001
GO:0055085	transmembrane transport	e	56/846	1.09E-06	<0.001
GO:0044710	single-organism metabolic process	e	119/846	8.21E-06	0.002
GO:0090304	nucleic acid metabolic process	p	18/846	3.19E-05	0.004
GO:0006396	RNA processing	p	1/846	5.71E-05	0.01
GO:0046483	heterocycle metabolic process	p	31/846	6.80E-05	0.01
GO:0072350	tricarboxylic acid metabolic process	e	7/846	7.88E-05	0.012
GO:1901360	organic cyclic compound metabolic process	p	33/846	0.00011	0.024
GO:0006139	nucleobase-containing compound metabolic process	p	27/846	0.00013	0.032
GO:0034641	cellular nitrogen compound metabolic process	p	42/846	0.00013	0.032
GO:0016070	RNA metabolic process	p	11/846	0.00017	0.046

1 Enrichment (e) or purification (p) detected. Enrichment indicates that the GO category is more highly represented than expected by chance and purification indicates that the category is underrepresented.

2 Number of differentially expressed genes in this category compared with total differentially expressed genes.

3 Adjusted p-value of enrichment or purification after Benjamini-Hochberg FDR correction.

## Discussion

We determined how parasitism affects the expression patterns of *P. destructans* genes by comparing expression levels between the fungus in culture and during host infection. We used dual RNA-Seq data and an approach that simultaneously mapped the reads to both host and pathogen transcriptomes followed by the removal of reads that mapped to host transcripts. This approach allowed for the estimation of expression levels of *P. destructans* genes with high levels of confidence by using RSEM to control for the uncertainty of multi-mapped reads. We compared gene expression changes of the cultured 20631–21 North American strain of *P. destructans* to infection of a naïve North American species. Although the data set had limited read depth for *P. destructans* genes in the *M. lucifugus* samples, we observed significant differential gene expression in 211 genes, or 2.2% of the 9575 known *P. destructans* genes. This initial study has validated this approach to identifying changing patterns of pathogen gene expression. Future studies will be needed to overcome some of the limitations of the currently available data sets by using greater read depth for the dual RNA-Seq data, better matching environmental conditions *in vitro* to those in hibernacula, and using the identical isolate of *P. destructans* for both data sets. Future work could also compare changes in *P. destructans* gene expression during infection of North American or European bat species that show more resistance to WNS mortality than *M. lucifugus*.^8,19,20,33,34^

As expected, we found that the transition from abiotic to parasitic growth was accompanied by many changes in *P. destructans* gene expression. Differences in temperature and humidity could also contribute to the differences in gene expression that we observed. Some of the gene expression changes are also presumably due to alterations in nutrient availability, such as the increased expression of lipase (*VC83_00616) in vivo* due to the high lipid content of mammalian skin. Although the cultured *P. destructans* was not grown on the same substrate that it would find in the environment, many of the gene expression changes that we observed appear consistent with adaptation to the host environment, rather than changes due to nutrient sources. For example, the increased expression of heat shock genes is consistent with the response to arousal from torpor to euthermic body temperatures that occurred 60 to 120 minutes before collecting the *M. lucifugus* samples.^27^ Correspondingly, a single sample from a bat that was allowed to become euthermic only briefly did not show upregulation of *P. destructans* heat shock genes (unpublished results). Thermal stress caused by a febrile response in the human host has been shown to activate a heat shock response in *Candida albicans*, preventing deleterious protein unfolding and aggregation.^35^ This heat shock response could be important for fungal survival in our system, as bats arouse to euthermic temperatures several times throughout hibernation (thus several times throughout *P. destructans* infection), and susceptible populations arouse from torpor more frequently during WNS.^13,14,36^

Consistent with a response of the pathogen to evade host immune recognition, we also found large increases and decreases in expression of genes involved in fungal cell wall structures (Table 2). The fungal cell wall is composed of an inner layer of chitin, a middle layer of β-glucans, and an outer layer of mannose. The cell wall provides rigidity and structure, however is also highly dynamic. The pattern recognition receptor Dectin-1 has been shown to be a receptor for fungal 1,3-β glucans and 1,6-β glucans,^37,38^ thus cell wall components serve to alert the mammalian immune system of a fungal pathogen. We have observed that Dectin-1 and several other C-type lectin domain family members are significantly upregulated in bat tissues infected with *P. destructans*.^27^ Consistent with this host observation, we detected significant alterations in *P. destructans* enzymes predicted to be involved in fungal cell wall remodeling (Table 2). *VC83_00788* and *VC83_04729*, homologs of Endochitinase 1, an enzyme which randomly cleaves and breaks down chitin, are upregulated 11.6 and 6.3-fold, respectively, while *VC83_05104*, a homolog of Chitin synthase 4 is downregulated 3.4-fold in *P. destructans* during infection compared with culture. Two homologs to Glucan endo-1,3-β glucosidases were differentially regulated; *VC83_07327* was upregulated in *P. destructans* during infection while *VC83_09076* was upregulated during culture. These enzymes presumably regulate cell wall β-glycan turnover and catabolism of β-glycans^39^ by removal of non-reducing terminal glucosyl residues from saccharides and glycosides.

Additionally, 3 Mannan endo-1,6-α mannosidases that were differentially expressed between *P. destructans* actively infecting a host and growing in culture (Table 2). Two were upregulated in culture (*VC83_00261* and *VC83_01650*), and one was upregulated during WNS (*VC83_07145*). Mannan endo-1,6-α mannosidases are required for normal synthesis of the cell wall and alkaline pH-induced hypha formation, as well as being responsible for random hydrolysis of α-mannosidic linkages in unbranched mannans.^40^ It is likely that the changes in Glucan endo-1,3-β glucosidase and Mannan endo-1,6-α mannosidase gene expression that we observed upon the switch from abiotic growth to host colonization leads to substantial alterations in the cell wall structures. The resulting differences in saccharide and glycoside branching patterns in the cell wall could make the pathogen less recognizable to the mammalian immune system.

Alternatively, these changes in cell wall enzyme gene expression could be due to changes in metabolic pathways that accompany the shift from abiotic to infectious niches. Different carbon sources can modulate cell wall structure and virulence in *C. albicans*.^41,42^ It is possible that changes in cell wall structures are caused by differences in metabolism when infecting bats, rather than direct adaptation to the host.

Alterations in cell wall structures also accompany shifts in the morphological growth type of fungi, such as a shift from yeast to hyphal phase in *C. albicans*.^37^ However, *P. destructans* grows vegetatively as hyphae on both Sabouraud's dextrose agar medium in culture,^43,44^ and when forming cupping erosions in the wing tissue of the host.^12,19^ Thus there is no difference in morphotype between our cultured and WNS *P. destructans* samples that might explain the dramatic alterations in expression of cell wall remodeling enzymes that we observed. Consequently, we propose that changes in the β−glucan landscape on the fungal surface via cell wall remodeling are a mechanism of immune evasion for *P. destructans*, similar to other fungal pathogens.^45^

Alterations of the cell wall during infection could explain the ineffectiveness of antibodies that recognize the cell wall of cultured *P. destructans* in providing protection from WNS.^46,47^ These results may also explain why immunization with either cultured *P. destructans* or a β-glucan vaccine^48^ did not affect the susceptibility of *M. lucifugus* to WNS (J. Johnson, J. McMichael, D. Reeder, and K. Field, unpublished). The antigens provided by these immunizations may not be present on the surface of *P. destructans* during infection because of changes in the cell wall structure that accompany the transition from abiotic to parasitic growth.

Because tissue invasion is a hallmark characteristic of *P. destructans* infections during WNS, we expected that expression of genes involved in degradation of the extracellular matrix would be upregulated. Unexpectedly, we found that the previously characterized subtilase-family of secreted proteases^30,31^ showed lower expression in *P. destructans* during infection than in culture. Instead, the homolog of the *A. fumigatus* vacuolar protease, major allergen Aspf2, showed high levels of expression during infection of *M. lucifugus* and was significantly upregulated compared with culture conditions. This suggests that other proteases may be better targets for preventing colonization than the subtilase-family proteases, although the possible role of Aspf2 in tissue invasion remains unknown. It is also plausible that subtilase-family proteases are regulated at a post-transcriptional level or are used by the fungus primarily during initial colonization. Therefore, further proteomic and expression time-course experiments may prove useful to further dissect the infection. Nevertheless, the abundant expression of *Aspf2*, known to be an *A. fumigatus* allergen in humans,^49^ suggests that further investigation of IgE-mediated allergic reactions during WNS may be warranted.

Infection of hosts was also associated with changes in expression for several genes involved in the transport or homeostasis of metal ions, including zinc, iron, and copper. This fungal response may be due to limited availability of some of these micronutrients in the host, which is likely sequestering metal ions as a form of nutritional immunity.^27,50^ Changes in micronutrient acquisition gene expression appear to be associated with host colonization, including increased expression of the zinc transporter *Zrt1*, the copper homeostasis factor *ATX1*, and a putative copper transporter, as well as the unexpected loss of siderophore import using *MirB*.^51^ Homeostasis of these micronutrients is essential for normal fungal metabolism and for the ability of the pathogen to respond to the oxidative stress activated by the host immune response.^50^ However, our gene ontology analysis (Table 4) indicates that genes involved in oxidation-reduction pathways are more highly expressed during growth in culture than host colonization. Enrichment of pathways involving peptide metabolism and translation in *P. destructans* infecting bats (Table 4) indicates that host colonization demands higher levels of protein expression than abiotic growth. Competition between the host and pathogen for micronutrients and the generation of oxidative stress likely varies over the course of infection^52^ and further study is needed to dissect this time course.

**Figure 4.**
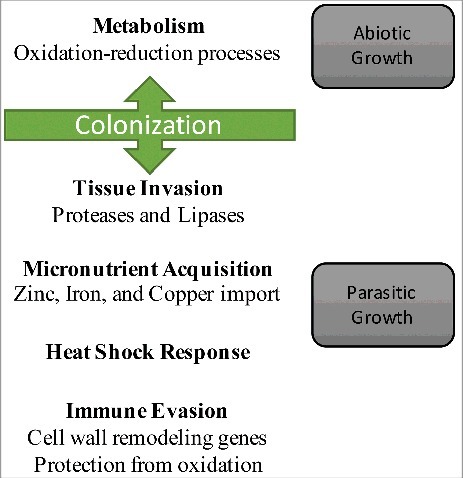
Model of the *P. destructans* gene expression changes that accompany WNS. Gene expression changes by *P. destructans* are compared for abiotic and parasitic growth. The changes in gene expression that we found are associated with these phases are indicated.

Together, these results provide a model of gene expression changes in *P. destructans* that accompany the transitions from abiotic to parasitic growth (Figure 4). This model provides a framework to understand how the pathogen responds with phenotypic plasticity to the environment and its host to adopt a virulent phenotype. Our results also suggest approaches to minimize virulence and/or colonization by targeting immune evasion, micronutrient acquisition, tissue invasion, or the heat shock response. Efforts to understand why some species are more susceptible to WNS than others will require further examination of host-pathogen interactions to determine if the pathogen responds differently in hosts that exhibit lower WNS susceptibility.

## Materials and methods

### Sample collection

Two different data sets were used for this study (Table 1). The samples for the first data set (MyLu) consisted of wing tissue from 6 individual *P. destructans-* infected *M. lucifugus* (little brown myotis) collected 60–120 minutes after arousal to euthermy from hibernation from caves in Kentucky, USA, as described previously.^27^ Hibernacula temperatures were 4–6 C at the time of collection and, based on our previous experience, we estimate that skin temperature varied between 4 and 8 C during torpor and up to 37 C during periodic arousals. The second data set was obtained from the North American 20631–21 strain of *P. destructans* growing in culture by D. Akiyoshi and A. Robbins (Department of Infectious Disease and Global Health, Cummings School of Veterinary Medicine, Tufts University). The 20631–21 strain of *P. destructans* was obtained from D. Blehert (National Wildlife Health Center, US. Geological Survey, Madison, WI, USA). The fungus was grown in culture at 10–14°C for 23 d on Sabouraud dextrose agar plates (BD Diagnostics, #221180) (Table 1). Sabouraud dextrose agar contains nutrient sources of dextrose, pancreatic digest of casein, and peptic digest of animal tissue. RNA was isolated using a Qiagen RNeasy Lipid Tissue Kit after disruption of the cells using Zymos BashingBead Lysis Tubes and a bead beater on maximum speed for 30 sec for 3 times and then 20 sec once, with cooling on ice between each.

### RNA sequencing

RNA sequencing was performed using Illumina sequencing as summarized in Table 1. Prior to analysis all data sets were quality trimmed using Trimmomatic v.0.35^53^ with the parameters SLIDINGWINDOW:4:5 LEADING:5 TRAILING:5 MINLEN:25. For samples with paired-end sequencing, only reads with both pairs remaining after trimming were used for further analysis. Analysis of the reads using FastQC v0.11.5^54^ and the results of STAR mapping indicate that there are no significant differences in the quality of the RNA in any of the cultured samples from the MyLu samples.

### Differential expression analysis

The quality trimmed reads were aligned using STAR v.2.5.1b^55^ to the concatenated genomes of *M. lucifugus* and *P. destructans*. For *M. lucifugus*, we used genome assembly Myoluc2.0 and gene models from Ensembl release 84.^56^ For *P. destructans*, we used the genome assembly and gene models from Drees et al..^57^ RSEM v1.2.29^58^ was then used to apply an expectation maximization algorithm to predict gene expression counts for each transcript. The expected count matrix for all samples is available in Data Set S1. To determine if the number of reads mapped to *P. destructans* transcripts provided sufficient statistical power to detect differential expression of these genes, we used Scotty^59^ to analyze the expected counts generated by RSEM. We determined that 65% of *P. destructans* genes expressed at a minimum of 4-fold change could be detected with a *p*-value cutoff of 0.05. Transcripts per million (TPM) was calculated by normalizing read counts for the length of each transcript and adjusting for the library size of mapped reads for each sample.^58^ The *M. lucifugus* transcripts were then removed from the analysis and differential expression was determined using only *P. destructans* transcripts.

Differential expression between conditions was determined using either DESeq2 v1.10.1^60^ or edgeR v.3.12.1^61^ after normalizing across samples using the trimmed mean of M-values (TMM) method^62^ and a minimum expression level of 2 TPM combined across all samples. False discovery rate (FDR) was used to control for multiple comparisons using the Benjamini-Hochberg procedure.^63^ Hierarchical clustering was performed using R stats package v3.3.1 with Pearson correlation complete-linkage clustering of Euclidean distances. Clustering was confirmed by bootstrap analysis using pvclust v2.0–0^64^ at an α level of 99% and 100 000 iterations. Genes without expression (expected count < 1) in at least 2 MyLu samples were excluded from the final analysis. Annotations for each gene were determined by using Trinotate v3.0, NCBI BLAST v2.2.29+^65^ with the UniProtKB/SwissProt database (E-value cutoff of 1 × 10^−4^), and InterProScan v.5.20–59.0.^66^ Gene ontology annotations were extracted from the InterProScan results and gene ontology enrichment analysis was performed using GOATOOLS v0.6.9^67^ with enrichment or purification measured by Fisher's exact test after FDR correction.

## Supplementary Material

1342910_supp.zip
