# Supplementary material for: Pseudogymnoascus destructans transcriptome changes during white-nose syndrome infections
Source: Virulence. 2017 Jul 13;8(8):1695–707. doi: 10.1080/21505594.2017.1342910 (PMC5810475; doi:10.1080/21505594.2017.1342910)
Supplement: 1342910_supp.zip [file kvir-08-08-1342910-s001.zip › 1342910_supp/2016VIRULENCE0371R1-s04.docx]

**Supplementary Table 1.** Transcripts per million mapped reads (TPM) for each sample after using STAR and RSEM to assign reads to the combined *M. lucifugus* and *P. destructans* transcriptomes.

| Group | Sample | *P. destructans* TPM |
| --- | --- | --- |
| Uninfected *M. lucifugus* | SSD011MYUN | 15 |
|  | SSD064MYUN | 14 |
|  | SSD075MYUN | 155 |
|  | SSD090MYUN | 47 |
|  | SSD114MYUN | 21 |
| Wild-infected *M. lucifugus* (MyLu) | KYMYLU06W | 13 985 |
|  | KYMYLU07W | 17 336 |
|  | KYMYLU11W | 14 719 |
|  | KYMYLU19W | 24 341 |
|  | KYMYLU23W | 20 126 |
|  | KYMYLU39W | 13 082 |
