# Supplementary figures and images for: Pseudogymnoascus destructans transcriptome changes during white-nose syndrome infections
Source: Virulence. 2017 Jul 13;8(8):1695–707. doi: 10.1080/21505594.2017.1342910 (PMC5810475; doi:10.1080/21505594.2017.1342910)

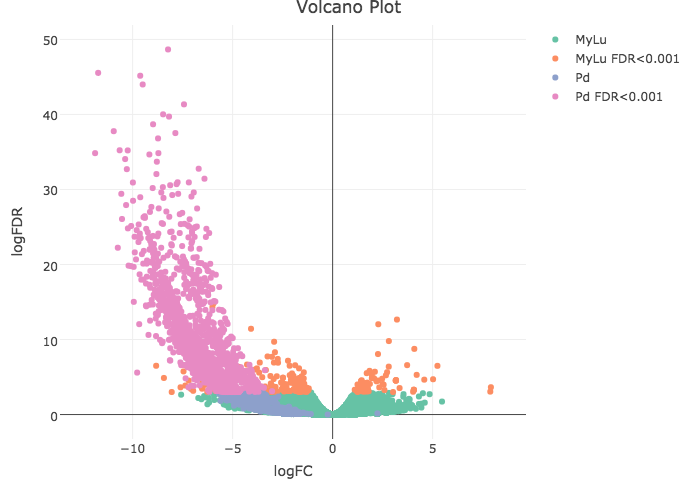

Supplement: 1342910_supp.zip [file kvir-08-08-1342910-s001.zip › 1342910_supp/2016VIRULENCE0371R1-s02.tif]
